# EEG-Based Personal Identification by Special Design Domain-Adaptive Autoencoder

**DOI:** 10.3390/s25206457

**Published:** 2025-10-18

**Authors:** Muhammed Esad Oztemel, Ömer Muhammet Soysal

**Affiliations:** 1 Division of Electrical and Computer Engineering, Louisiana State University, Baton Rouge, LA 70803, USA; moztem2@lsu.edu; 2 Computer Science, Southeastern Louisiana University, Hammond, LA 70402, USA

**Keywords:** biometric, fingerprint, brainprint, EEG, identification, autoencoder, domain generation

## Abstract

Individual brain activity patterns derived from electroencephalogram (EEG) data offer a unique source for personal identification, introducing a novel approach to the field. Autoencoders are well-known machine learning models that automate feature extraction, which is a crucial step in biometric identification. Among various types of autoencoders, the domain-adaptive autoencoder (DAAE) is explored for feature extraction. The extracted latent features are employed by four machine learning classifiers, KNN, ANN, SVM and RF, for personal identification. Two domain adaptation approaches were presented. The proposed frameworks were evaluated in a longitudinal setting, using three types of EEG recordings: resting state, auditory and cognitive stimuli. Model performance was assessed through experiments involving seven-, five- and two-subject classification tasks. The highest identification accuracy, 100%, was achieved by the SVM-based model in the two-subject experiment, using features extracted with the uniform referential DAAE. Similarly, the RF-based model attained an accuracy of 99.84% in the two-subject experiment when trained on features obtained from the softmin referential DAAE. As expected, accuracy declined with an increasing number of subjects in the dataset, reflecting the difficulty of multi-subject classification.

## 1. Introduction

EEG-based subject identification has gained increasing attention in recent years, due to its potential for secure and non-invasive biometric systems. Traditional biometric methods like fingerprint, iris scan and face recognition systems have been used for personal identification. However, these methods have been associated with various security and privacy concerns [1]. In contrast, an EEG signal reflects internal neural dynamics which are inherently unique to everyone. The complexity of these neural signatures makes them exceptionally difficult to replicate and provides a robust defense against malicious attempts to compromise biometric security systems [2]. In addition, EEG signals are not directly observable through other biometric modalities, offering an additional layer of protection. Although EEG-based biometric systems provide more secure subject identification, the non-stationary nature of EEG signals introduces challenges in feature extraction and generalization across data which are collected in separate sessions.

Traditional feature-extraction methods, such as fast Fourier transform, auto regressor, power spectral density and wavelet transform, have been extensively employed in EEG-related research, including brain–computer interface applications [3], distinguishing neurological disorders [4], emotion recognition [5] and EEG-based biometric applications [6]. Al-Fahoum et al. highlighted the limitations of traditional feature-extraction methods in EEG-related research, such as computation time, manual parameter tuning and accurate spectral estimation [7].

Deep learning algorithms are capable of capturing non-linear relationships and automatically extracting meaningful, hierarchical features within EEG data, eliminating the laborites feature-engineering requirement [8]. While a convolutional neural network (CNN) is well suited for capturing spatial features [9], a recurrent neural network (RNN), particularly long short term memory (LSTM) or gated recurrent unit (GRU), are designed to extract temporal features and sequential patterns [10]. CNN and RNN have been effectively used for feature extraction from EEG data either independently or hybrid architecture to perform comprehensive feature extraction in EEG-based biometric research [11,12].

In addition to these established architectures, an autoencoder (AE) offers a unique approach for feature extraction through unsupervised learning. An AE aims to encode input data into a lower dimensional latent representation and then decode it back, to reconstruct the original as accurately as possible. In training the model, an autoencoder performs error calculations and utilizes backpropagation, similarly to traditional neural network architectures. AEs have been used for EEG-related research, including anomaly detection [13], dimensionality reduction [14], signal reconstruction [15] and feature extraction [16].

While deep learning approaches, including autoencoders, offer significant advantages for automation and feature learning capabilities, it is important to acknowledge that training complex architectures, such as 3D convolutional autoencoders, requires substantial computational resources and high-performance GPUs. This computational intensity represents a trade-off where the primary benefits lie in eliminating manual feature engineering, improving automation and potentially enhancing robustness across different datasets, rather than in computational efficiency during the training phase. However, once trained, these models can provide efficient inference for real-time applications, making the initial computational investment worthwhile for scenarios requiring robust, automated feature extraction.

A regular autoencoder utilizes the same target domain that can be generated by means of a self-referencing approach. On the other hand, for the cases where the target domain is not the same as the source domain, the domain-adaptive autoencoders (DAAE) are specialized in order to align the feature distribution between encoder and decoder domains [17] for extracting distinct latent features. DAAE are used for emotion recognition [18] and cross subject motor imagery classification [19]. Among domain adaptation approaches, the sample hybridization or synthetic augmentation has been shown to enhance feature representation and improve class separability. Shadi et al. demonstrated an EEG-based domain-adaptation framework, where samples from the target domain, aligned with source distributions, led to improved intra-class cohesion and cross-domain generalization [20].

Spatial representations of EEG data, such as topographic maps, enable detailed visualization and analysis of regional brain activity [21]. By stacking these maps over time, it is possible to construct EEG data cubes, which encode both spatial and temporal information. These data cubes can be processed with 3D convolutional kernels, allowing 3D convolutional autoencoders (3D-CAEs) to learn volumetric features, which are particularly useful for subject identification tasks.

To assess the discriminative capability of the features extracted via domain-adaptive autoencoders, we employ a range of machine learning classifiers: K-Nearest Neighbors (KNN), Random Forest (RF), Support Vector Machines (SVM) and Artificial Neural Networks (ANN). These models are trained on features extracted from EEG data cubes and evaluated under both cross-session and cross-domain scenarios to measure robustness and generalizability.

A key challenge in EEG-based biometrics is the longitudinal variability of neural signals, which fluctuate due to mental state, task, environmental conditions and fatigue. Recognizing this, Gonzalez et al. developed the BED dataset, which includes EEG recordings from multiple sessions, enabling realistic evaluation of cross-session performance [22]. Relying solely on within-session data can lead to a significant overestimation of a model’s practical identification capabilities [23].

In this study, we propose a novel domain-adaptive autoencoder-based framework utilizing two referencing schemes, a uniform-weighted signal and a softmin-weighted signal, for EEG-based subject identification, using EEG data cubes. The proposed process generates two new domains. During the subject identification stage, the machine learning classifiers such as ANN, KNN, SVM and RF employ the latent features generated by the proposed autoencoder network. In the exploration of multi-subject performance of the framework, first, the system is tested over seven subjects. An identifiability analysis is conducted to find out the two least identifiable subjects. Following the removal of these two subjects, a new model is trained and evaluated with the remaining five subjects to investigate the effect of features extracted from so-called challenging subjects. In addition, a pairwise classification operation is conducted to investigate subject identifiability for each individual.

To the best of our knowledge, this is the first study to apply domain-adaptive autoencoders to EEG data cubes for biometric identification purposes. The remainder of this paper is organized as follows: Section 2 reviews related work; Section 3 details the proposed domain-adaptive framework methodology; Section 4 describes the experimental setup; Section 5 presents the results and evaluation metrics; and Section 6 concludes with a discussion and future directions.

## 2. Previous Work

EEG-based personal identification has been an active area of research for over two decades [1]. Various data collection protocols have been developed for EEG-based biometric studies, including resting-state recordings, responses to external stimuli (e.g., auditory cues), and mental task engagement. In resting-state protocols, participants are typically relaxed and free from cognitive or emotional activity [24], whereas external stimuli and mental task protocols involve specific activities designed to trigger the brain to generate responses [25,26].

Marcos et al. investigated EEG-based biometric identification using both resting-state recordings and task-induced EEG with visual and auditory stimuli. The time–frequency features were extracted using Continuous Wavelet Transform (CWT) across five frequency bands: delta (0.5–4 Hz), theta (4–8 Hz), alpha (8–13 Hz), beta (13–30 Hz) and gamma (30–50 Hz). Their findings revealed a substantial performance decline in cross-session identification accuracy, dropping to approximately 72–85% from the peak accuracies of around 99%, achieved when both training and testing datasets originated from the same session. These results emphasize the advantages of stimulus-evoked EEG for biometric applications, while simultaneously highlighting the persistent challenge of maintaining longitudinal consistency [27].

Traditional feature-extraction methods have several limitations, motivating researchers to seek more effective feature representations. In [28], the classification performances of various machine learning models were compared using both statistical features and features extracted via an autoencoder (AE) for subject identification. The results demonstrated that AE-derived features yielded superior classification performances compared to statistical features. Das et al. employed an autoencoder to extract latent features for EEG-based subject identification. The extracted features were subsequently processed by convolutional neural network (CNN) and long short-term memory (LSTM) architectures to determine the subject identity [29]. Similarly, Zhang et al. proposed an autoencoder architecture integrated with an attention module for analyzing Delta-band EEG signals from both their own collected dataset and the publicly available EEG Motor Movement/Imagery Dataset (EEGMMIDB). The learned deep features were then classified using an extreme gradient boosting (XGBoost) classifier to perform subject identification [30].

Domain-adaptive autoencoders (DAAEs) have recently gained attention in EEG research as an effective means of addressing the well-known issue of a domain shift, which arises from inter-subject, inter-session, or device-related variability. By integrating autoencoder-based feature learning with domain adaptation strategies, DAAEs enable the extraction of compact and noise-robust latent representations that remain invariant across domains, thereby improving the transferability of models trained on one subject or session to others. The key advantages of using DAAEs in EEG analysis include their ability to compress and denoise raw EEG signals to suppress artifacts and irrelevant variability, align the distribution of source and target domains in latent space to minimize cross-domain discrepancy, reduce reliance on labeled target data by leveraging unsupervised or semi-supervised adaptation and enhance robustness and generalization in downstream tasks, such as classification, seizure detection, or emotion recognition. Reference [31] introduced a subspace alignment autoencoder for emotion recognition, demonstrating improved cross-subject and cross-session generalization compared to non-adaptive baselines. Similarly, Wang et al. proposed a multi-modal domain-adaptive variational autoencoder to boost cross-domain emotion recognition accuracy [32]. More recently, Liu et al. employed an LSTM-autoencoder with domain adaptation to achieve subject-invariant EEG compression [33], while Peng et al. demonstrated that autoencoder-based domain adaptation can significantly enhance seizure prediction performance across patients [34]. Collectively, these studies highlight the effectiveness of DAAEs in overcoming domain variability and ensuring that EEG-based models remain reliable and generalized in real-world applications. However, to the best of our knowledge, the utilization of domain-adaptive autoencoders for EEG-based personal identification has not yet been explored in the literature, highlighting a key research gap that our study aims to address.

## 3. Methods

**Figure 1:**
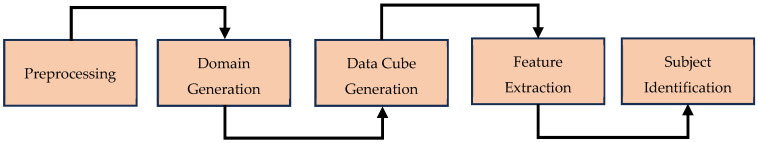
Proposed frameworks modules.

This section explains the proposed framework for EEG-based subject identification through DAAE. The framework consists of five key modules: preprocessing, target domain generation, EEG data cube generation from topomaps, feature extraction using DAAE and subject identification using SVM, RF, KNN and ANN. Figure 1 illustrates the overall pipeline.

### 3.1. Preprocessing

**Figure 2:**
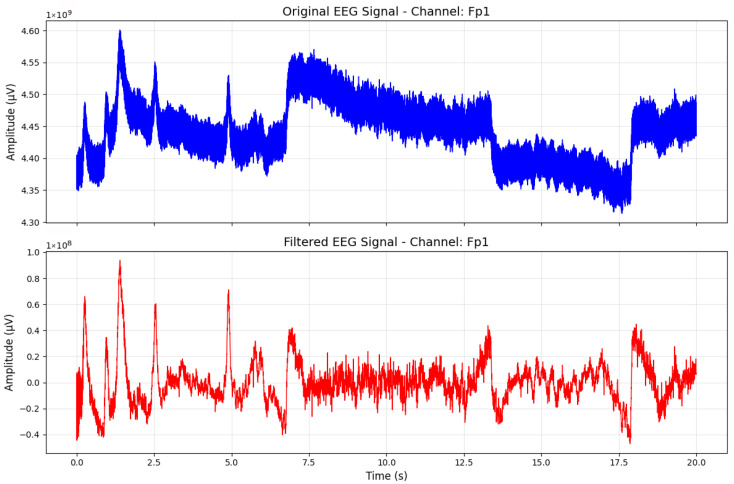
A preprocessed signal.

In the preprocessing stage, notch filters were applied to eliminate power line interference and its harmonics at 60 Hz, 120 Hz, 180 Hz and 240 Hz from the EEG signals. A notch filter [22] reduces the narrow frequency band while allowing all other frequencies to pass. After the removal of line harmonics, a high-pass filter with a 0.5 Hz cutoff was applied to remove slow baseline drifts and low-frequency physiological artifacts. Following this, the EEG signals were band-pass filtered into eight distinct frequency ranges: Delta (1–4 Hz), Theta (4–8 Hz), Alpha (8–13 Hz), Beta (13–32 Hz), Delta–Beta (De2Be: 1–32 Hz), Theta–Beta (Th2Be: 4–32 Hz), Gamma (32–125 Hz) and All (>1 Hz). Figure 2 demonstrated the preprocessed EEG signal for a single channel signal.

### 3.2. Target Domain Generation

A domain-adaptive autoencoder is a specialized autoencoder where the encoder and decoder receive signals from related but different domains, allowing the model to learn transformative features. During the reconstructing process, the decoder is guided to produce a reference target that is from a different domain than the source domain. The autoencoder’s job is to learn how to reconstruct the weighted-referential signal from an individual signal that is used to compose the target signal. In other words, the ‘common’ latent patterns for a subject are generated by the encoder in such a way that the decoder is forced to learn how to make the source signal mimic the target signal.

**Figure 3:**
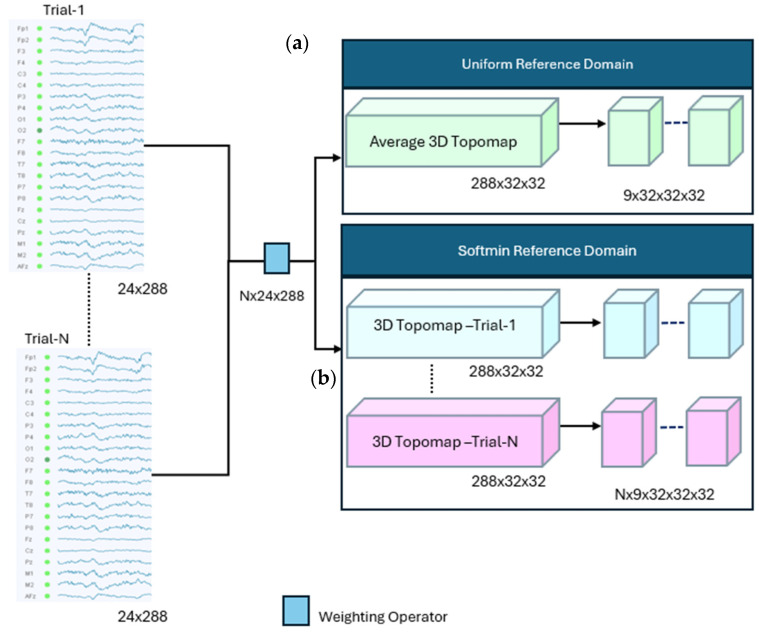
Target domain generation, (**a**) uniform reference-based domain, (**b**) softmin reference-based domain.

In this study, we utilize evoke potential-based target domain generation by means of two weighting schemes, namely uniform-based and softmin-based weighting. The shift in the target domain allows the autoencoder to learn evoked potential patterns that can provide a distinct feature representation. Figure 3 illustrates the proposed process for generating the target domain from multiple EEG trials using both the uniform and softmin reference signals.

The uniform reference-based approach assigns the same weight to each trial. The softmin reference-based approach assigns a weight to its trial at any moment, based on the similarity of the signal of the corresponding trial to the reference signal that is obtained in all trials. The reader can observe that the softmin scheme can generate more specific context-aware features compared to that of the uniform scheme. In the following figure, the details of each approach will be presented.

#### 3.2.1. Uniform Reference-Based Signal Domain

The uniform reference-based autoencoder employed evokes potential signals obtained from multiple trials; a reference target signal that will be used by the decoder during the reconstruction is obtained by averaging signals over these trials.

The autoencoder is trained to minimize the reconstruction error, learning to extract features that represent the common patterns across trials. This approach aims to capture the average neural responses while reducing the intra-trial variability of the reference signal compared to the self-referential approach. The weighted average signal $S_{UR}\left(t\right)$ is obtained by Equation (2), where $\;W_{U}\left(t\right)$, *K* and $S_{i}\left(t\right)$ denote the uniform weight signal, the number of trials and the signal at the trial *i*, respectively. An average 3D topomap signal is generated from the reference signal $S_{UR}\left(t\right)$; following this, the data cube is composed from the 3D signal, as explained in the sequel. Figure 3a demonstrates the data cube generation from $S_{UR}\left(t\right)$.

(1) $$W_{U}\left(t\right)=\frac{1}{K}\;,\;$$

(2) $$S_{UR}\left(t\right)=\sum_{i=1}^{K}W_{U}\left(t\right)\;S_{i}\left(t\right),$$

#### 3.2.2. Softmin Reference-Based Signal Domain

The softmin operator adjusts the significance of neural activity $S_{i}\left(t\right)$, recorded at a trial *I*, based on its similarity to the reference signal $R\left(t\right)$, as described by Equations (3)–(5). The adjustment factor is determined by the similarity between a trial signal and the reference signal. A lower similarity produces smaller weight values, allowing us to suppress dissimilar sections of the signal in the construction of the target signal while keeping the most common segments.

The weight function $W_{S_{i}}\left(t\right)$ of a trial signal $S_{i}\left(t\right)$ evaluates the similarity between each individual trial and the reference signal. Since the exponential function decays as its argument increases, a smaller difference between an individual trial signal and the reference signal yields a larger weight value, assigning greater importance to the trial that closely resembles the reference. The parameter β acts as a sensitivity factor that controls the exponential function behavior. The $W_{S_{i}}\left(t\right)$ with a larger β behaves more sensitively to the deviation, rapidly reducing the weight assigned to a trial signal at the time *t*, according to its deviation from the reference, whereas a smaller β value results in a slower decay, thereby allowing greater tolerance to dissimilarity. Data cubes generation from $S_{SR_{i}}\left(t\right)$ is illustrated in Figure 3b.

The softmin reference-based target domain generation process is finalized by generating a 3D topomap signal for each individual trial from $S_{SR_{i}}\left(t\right)$. Figure 3b illustrates the data cube generation process.

(3) $$R\left(t\right)=median\left(S_{i}\left(t\right)\right)\;,\;\;\;$$

(4) $$W_{S_{i}}\left(t\right)=\frac{e^{-\beta \left|S_{i}\left(t\right)-R\left(t\right)\right|}}{\sum_{1}^{K}e^{-\beta \left|S_{i}\left(t\right)-R\left(t\right)\right|}},$$

(5) $$S_{SR_{i}}\left(t\right)=W_{s}\left(t\right)\;S_{i}\left(t\right)$$

### 3.3. EEG Data Cube Generation

EEG signals from multiple positions across the brain are simultaneously recorded. In the detection of distinct patterns, combining temporal and spatial information is crucial. The proposed topomap representation provides valuable insights for a more comprehensive analysis of brain signals. In the framework, the autoencoder’s source domain and target domains are designed in the form of topomaps.

**Figure 4:**
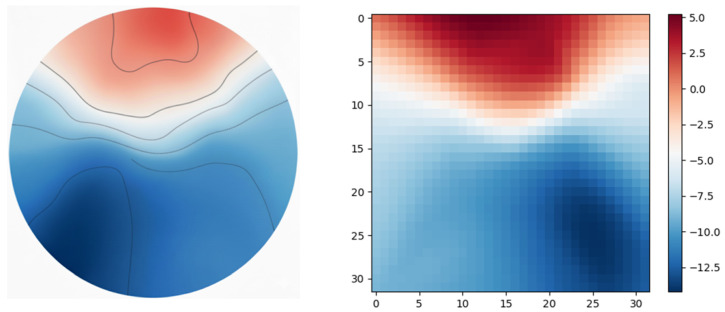
Spatial brain response.

Topomaps are generated by mapping the voltages recorded from EEG electrodes—arranged according to standard layouts, such as the International 10–20 system—onto a 3D spherical model of the head. These electrode positions are then transformed into a 2D plane using an azimuthal projection, which flattens the spherical surface while preserving spatial relationships from the vertex outward. The discrete voltage measurements are interpolated across the 32 × 32 grid, producing a smooth color-coded representation of potential distribution over the scalp. Figure 4 shows the spatial brain response and its corresponding grid version. 

By stacking sequential topographic map data over time, brain responses can be represented volumetrically, enabling the simultaneous capture of spatial and temporal dynamics in a single 3D structure. These volumetric representations can then be used in the feature extraction process to generate richer and more informative inputs for subsequent analysis or classification tasks.

**Figure 5:**
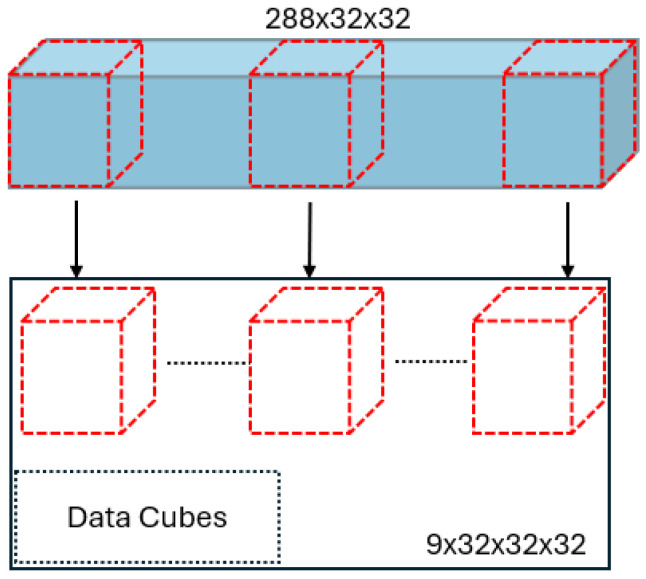
Data cube generation.

Each EEG recording spans 3 s, yielding 3000 samples. Due to minor channel- and trial-level data loss caused by irregular dropouts during acquisition, a total of 2880 data points were retained. The median of every 10 consecutive samples is computed, resulting in a down-sampled signal of 288 time points per EEG record. Extracting the central tendency of every 10 data points using the median, rather than the mean or simple decimation, provides robustness against transient artifacts and outliers that are common in EEG recordings, thereby preserving the representative trend of the signal while reducing the influence of noise. Applying these steps produces a 3D EEG data stream with dimensions of 288 × 32 × 32. This stream is then partitioned into nine non-overlapping segments for further processing. Figure 5 illustrates how data cubes with size of 32 × 32 × 32 are achieved from the 3D EEG data stream.

### 3.4. Feature Extraction with 3D-CAE

In this study, a three-dimensional convolutional autoencoder (3D-CAE) is employed for feature extraction, as it is particularly effective in modeling both spatial and temporal dependencies. Formally, the autoencoder comprises an encoder function $f_{\mathrm{enc}}$ that maps an input tensor $X\;\epsilon \;{\mathbb{R}}^{32\times 32\times 32}$ to a latent feature tensor *Ƶ* $\epsilon \;{\mathbb{R}}^{d\times d\times d}$, where *d* denotes the size of the dimension. The decoder function $f_{\mathrm{dec}}$ is trained to map the tensor *Ƶ* to the reconstructed data cube stream $\hat{X}$ of the target domain.

**Figure 6:**
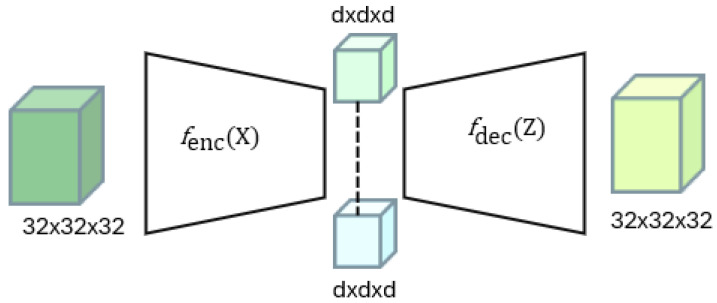
Feature extraction via autoencoder.

The training process optimizes the network by minimizing the reconstruction loss $L\left(X_{A},\hat{X}\right)$, defined as the mean squared error between the domain-shifted $X_{A}$ and its reconstruction $\hat{X}$, generated by the decoder. Note that the data cube $X_{A}$ is generated according to the process described in Section 3.2, Target Domain Generation. This optimization process enables extracting a compact and discriminative representation from the source domain, thereby reducing the reliance on manual feature engineering. Figure 6 illustrates the general outline of a 3D autoencoder process.

### 3.5. Subject Identification

A three-dimensional convolutional autoencoder (3D-CAE) is applied to EEG data cubes to derive compact latent representations that preserve the inherent spatial and temporal characteristics of the signals. The encoded features obtained from the final layer of the encoder—whose dimensionality depends on the number of units in that layer—serve as inputs for a subsequent classification stage. For each EEG data cube, the mean value of the extracted features is computed and provided to the classifiers. This two-stage framework leverages the unsupervised learning capability of the autoencoder to produce informative feature embeddings, thereby enhancing the performance of supervised classification models.

To evaluate the discriminative power and generalizability of the extracted features, four classification algorithms are employed: K-Nearest Neighbors (KNN), Artificial Neural Networks (ANN), Support Vector Machines (SVM) and Random Forests (RF). These classifiers were chosen to represent a broad spectrum of learning approaches, including instance-based, neural network-based, margin-based and ensemble-based methods. In addition, to assess cross-session consistency in EEG-based subject identification, a longitudinal evaluation is conducted, wherein each classifier is trained on data from Session 1 and tested on data from Session 2 that came at a later date.

### 3.6. Parameter Search

In this study, a Keras Tuner with Bayesian optimization is employed to identify the optimal set of hyperparameters for both the autoencoder and the classification models. For the autoencoder, the optimization objective is to minimize the reconstruction loss. In contrast, for the classifiers, the objective is to minimize classification loss by selecting the most effective hyperparameter configuration.

Bayesian optimization, a model-based search strategy, efficiently navigates the hyperparameter space by building a probabilistic surrogate model and selecting promising candidates based on prior evaluations. This approach is particularly advantageous for deep learning models, where training is computationally expensive. To evaluate the robustness and generalization capability of the proposed framework, a 5-fold cross-validation scheme is implemented. Within each fold, Bayesian optimization is conducted independently, tuning model hyperparameters based on the respective training–validation split. Table 1 presents hyperparameters and values.

## 4. Experimental Setup and Data

The EEG dataset was obtained from seven college student participants following approval from their university’s Institutional Review Board (IRB). All participants were college students above the age of 18, with normal stress levels and no diagnosed neurological disorders. None of the participants were taking medications that could affect neurological function at the time of data collection. Data collection took place over two sessions in spring 2023, 10 days apart from each other. EEG signals were recorded using an mBrain Train amplifier (sampling rate: 1 kHz), with a 24-channel headcap arranged according to the international 10–20 system, in conjunction with Neuro Behavioral Systems’ Presentation software (version 24.0 07.19.23). Each recording was labeled with a unique subject ID: sb106, sb328, sb330, sb381, sb455, sb717 and sb768.

To satisfy longitudinal study requirements, all training data were taken exclusively from Session 1, while testing data were obtained solely from Session 2, ensuring temporal separation between datasets. Models were trained using the high-performance computing facilities at Louisiana State University and Louisiana Optical Network Initiate (LONI), equipped with the NVIDIA A100 80GB PCI.

Models were trained using the high-performance computing facilities at Louisiana State University and the Louisiana Optical Network Initiative (LONI), which are equipped with NVIDIA A100 80GB PCI GPUs.

All experiments were implemented in Python (version 3.12), using the Visual Studio Code (version 1.105) environment. Data preprocessing and EEG signal handling were conducted with the MNE library, while scikit-learn was employed for machine learning utilities. Neural network models were developed with TensorFlow and Keras, and hyperparameter optimization was performed using a Keras Tuner to systematically explore model configurations and improve training performance.

Three stimulus types were employed: resting state, cognitive and auditory. In the resting state condition, participants closed their eyes and remained still for three seconds without performing any tasks. In the cognitive task, they engaged in inner speech, repeating the word “evergreen” for three seconds. For the auditory condition, participants listened to the sound of a conga drum for the same duration. These three types of stimuli were explored independently to examine their effectiveness for EEG-based subject identification. The resting state reflects intrinsic neural activity, the cognitive task elicits internally driven but reproducible responses, and the auditory task evokes externally stimulated brain activity. A total of 35 resting-state EEGs were recorded per session, and cognitive and auditory conditions were each recorded over 10 trials per session. All EEG trials lasted exactly three seconds. The dataset used in this study will be made available upon request.

In the uniform-based domain generation, a single 3D EEG signal is produced for all trials, whereas in the softmin-based domain generation, each trial is associated with its own corresponding 3D EEG signal, as described earlier in this study. During the calculation of weight values in Equation (4), the parameter *β* is set to 0.1.

## 5. Results and Discussion

**Figure 7:**
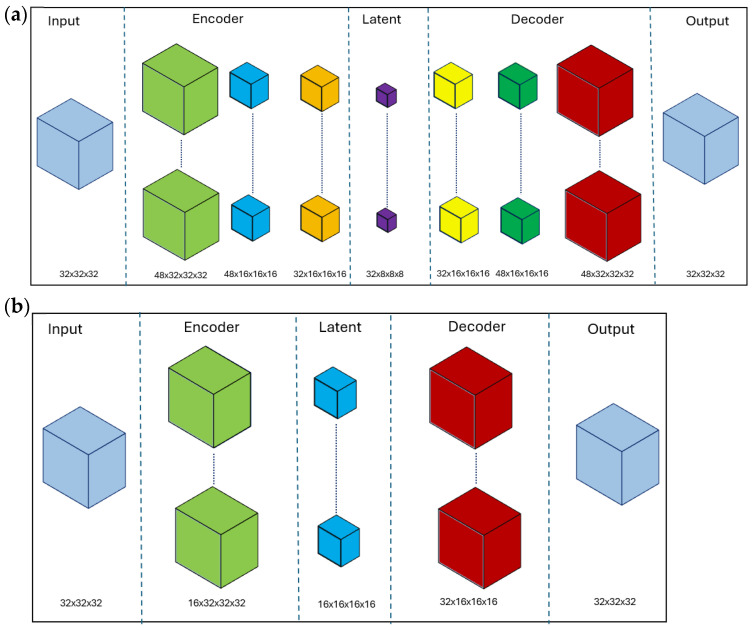
Autoencoder network structure, (**a**) uniform DDAE, (**b**) softmin DDAE.

As a result of the parameter search, the architecture of the autoencoders and the models used for subject identification were determined. The parameter search algorithm was executed independently for each stimulus and frequency band. In total, 48 network architectures were obtained, comprising 24 uniform DDAE and 24 Softmin DDAE models. For eight distinct frequency bands and three different stimuli, a total of 24 autoencoders were generated for each DDAE approach. Example autoencoder architectures for uniform DDAE and softmin DDAE are presented in Figure 7. Figure 7a illustrates the uniform DDAE network structure in the Gamma band for the resting-state stimulus, whereas Figure 7b illustrates the softmin DDAE network structure in the De2Be band for the auditory stimulus. The DDAE network structures shown in this figure represent the cases where the identifier achieved the highest performance for the corresponding approach.

Table 2 presents the selected parameters for both approaches, covering the autoencoders and the subject identification models.

**Figure 8:**
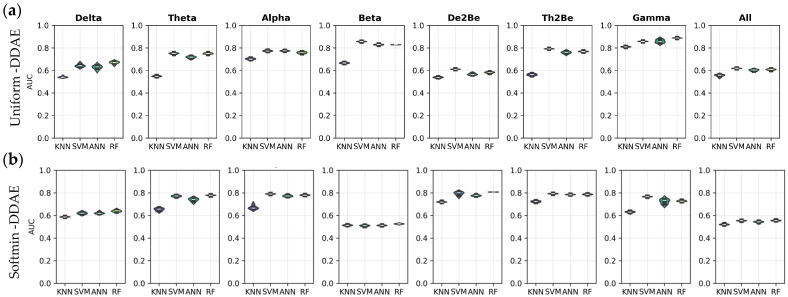
AUC performance of the models for resting state, (**a**) through uniform-DDAE features, (**b**) through softmin DDAE features.

Figure 8 compares the AUC performance of subject identification models using resting-state EEG data across eight distinct frequency bands. Figure 8a presents the classification results obtained from features extracted via the uniform DDAE approach, whereas Figure 8b shows the results derived from features extracted via the softmin DDAE approach. According to the results, the Random Forest-based model achieved the highest AUC performance in the Gamma band, with a mean score of 88.90% ± 0.0015, while the KNN-based model recorded the lowest performance within this frequency band. Overall, when comparing uniform DDAE and softmin DDAE, the models demonstrated superior subject identification performance when utilizing uniform DDAE features in the Beta band. Furthermore, it can be noted that the Gamma and Alpha bands consistently produced better results in both approaches when compared to the other frequency bands.

**Figure 9:**
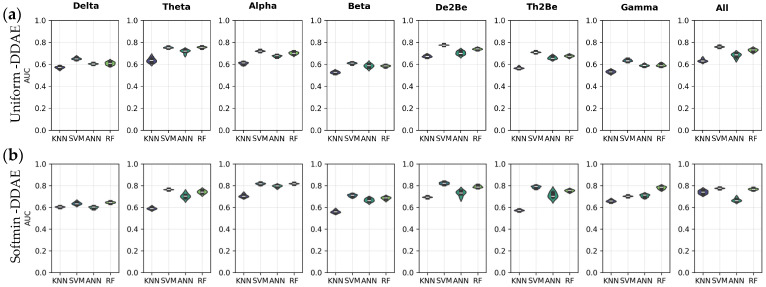
AUC performance of the models for auditory stimuli, (**a**) through uniform-DDAE features, (**b**) through softmin DDAE features.

Figure 9 compares the AUC performance of subject identification models based on features extracted from audiotory EEG data cubes using the uniform DDAE and softmin DDAE approaches. In general, the highest mean AUC performance 82.17% ± 0.0081 was achieved by the SVM-based model. This performance was obtained using features extracted from the softmin DDAE in the De2BE band. The performance of the models was evaluated across different frequency bands for both approaches, and the results were found to be relatively close to each other. Overall, all models exhibited a similar performance. However, in certain cases—such as the uniform DDAE condition in the Th2Be band—the KNN model demonstrated the lowest performance. In contrast, the SVM- and RF-based models generally achieved the highest performance.

**Figure 10:**
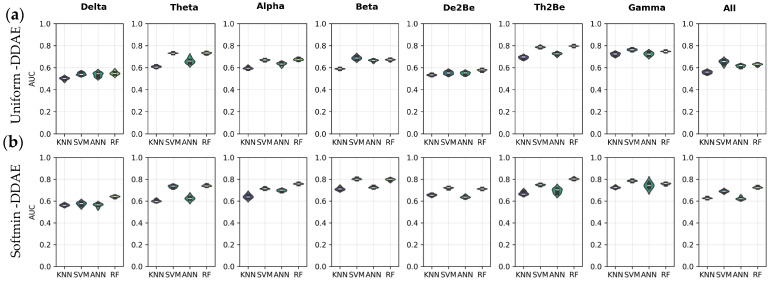
AUC performance of the models for cognitive task, (**a**) through uniform-DDAE features, (**b**) through softmin DDAE features.

Figure 10 provides a comprehensive comparison of the performance of subject identification models, based on features extracted from EEG data cubes associated with cognitive tasks using both the uniform DDAE and softmin DDAE approaches. The results indicate that model performance varies across different frequency bands. In particular, the Th2Be band yielded the highest mean AUC score 80.49% ± 0.0067 when using RF-based models through softmin DDAE features, highlighting this configuration as the most effective within that band. When comparing the two approaches more broadly, it was observed that while performances in certain bands were relatively close to each other, models utilizing softmin DDAE features in the De2Be band demonstrated superior subject identification performance. This suggests that softmin DDAE can provide more discriminative features for specific bands, thereby enhancing identification accuracy. Overall, both uniform DDAE and softmin DDAE offer distinct advantages, depending on the frequency band and the classification model applied, underscoring the importance of selecting the appropriate combination of band and feature-extraction method for optimal subject identification.

**Figure 11:**
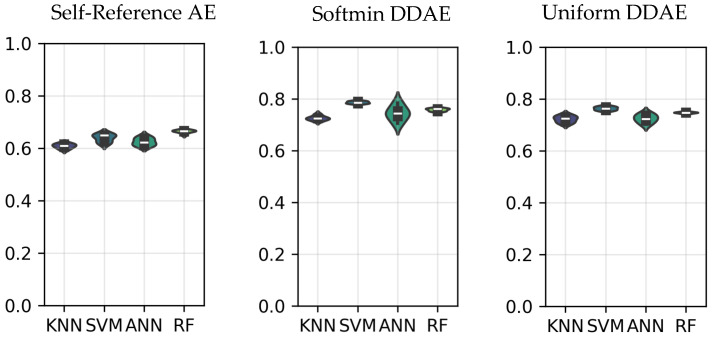
AUC performance for domain-based experiments.

Figure 11 presents a comparison of personal identification results based on features extracted from different domains. These performances were obtained from the Gamma band and cognitive-task-related EEG data cubes. As illustrated in the figure, all models achieved a superior subject classification performance to the case where the EEG data cubes were directly decoded without additional domain-based feature extraction, which is named self-referential AE [35]. DDAE provided a clear advantage by producing more discriminative subject-specific features, which in turn enhanced classifier performance. This improvement is evident in the results presented in Figure 11**.**

**Figure 12:**
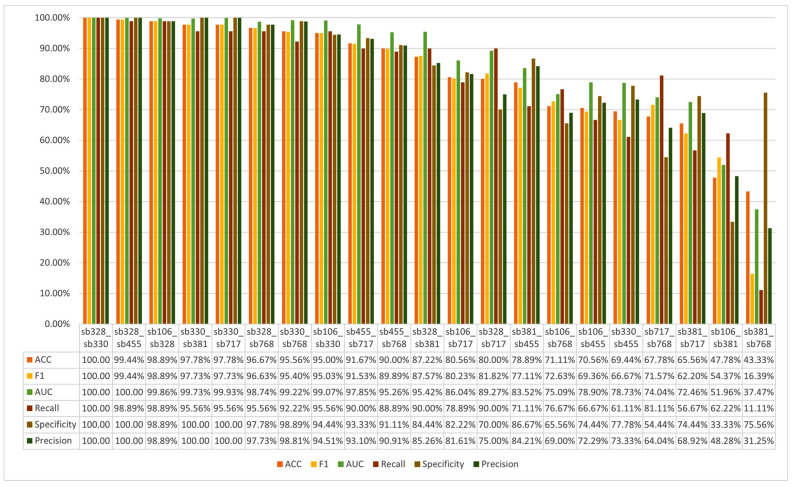
Pairwise identification through softmin-DDAE features.

Figure 12 presents the subject identification performance of the SVM-based model using features extracted from the softmin DDAE across all pairwise combinations of seven participants. The results reveal notable variations in the model’s ability to distinguish between different subject pairs. In particular, the combination of Sb328 and Sb330 were classified with perfect accuracy, indicating that the EEG features derived from these participants were highly discriminative and easily separable by the model.

In contrast, the combination of Sb381 and Sb768 exhibited the lowest classification performance. This suggests that the EEG features extracted from these two participants share a higher degree of similarity, thereby posing greater challenges for the model to achieve accurate separation. Overall, the majority of the pairwise classification tasks achieved an accuracy rate exceeding 70%, demonstrating that the SVM-based model, when using softmin DDAE features, possesses strong potential for reliable subject identification at the pairwise level. Furthermore, these results provide valuable insights into which subject pairs are more easily distinguishable, and which remain more challenging, offering a deeper understanding of the strengths and limitations of EEG-based biometric identification.

**Figure 13:**
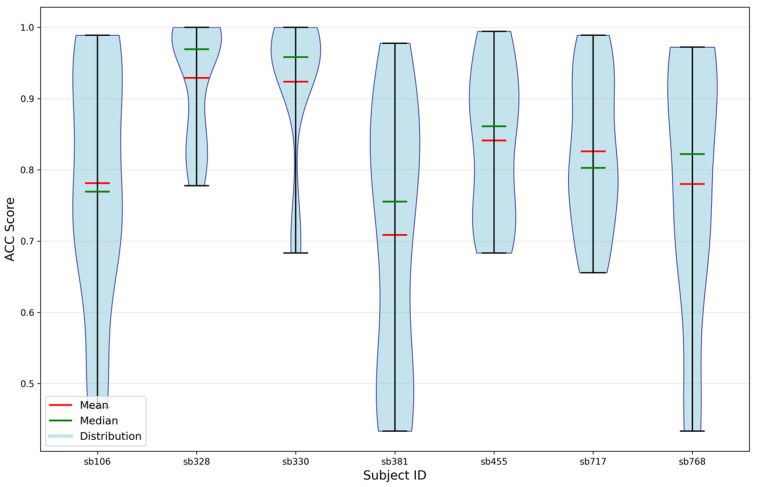
Subject identifiability through softmin DDAE features.

In addition, Figure 13 illustrates the subject identifiability when using features extracted from the softmin DDAE. These results were obtained through 5-fold cross-validation and represent the distribution of accurate identification across all pairwise combinations of participants. According to the findings, the least identifiable participants in this analysis were Sb106, Sb381 and Sb768.

**Figure 14:**
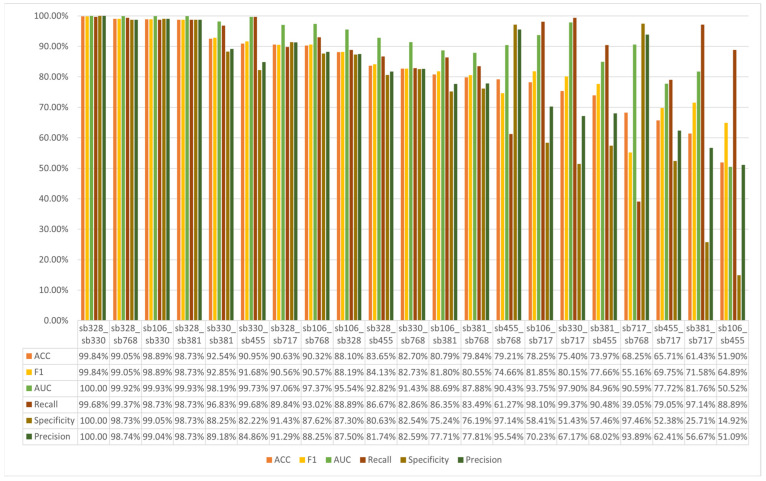
Pairwise identification through uniform DDAE features.

Figure 14 presents the pairwise subject identification outcomes of the RF-based model, utilizing features extracted from the Uniform DDAE across all possible combinations of the seven participants. The results highlight substantial differences in the model’s discriminative capacity across subject pairs. Notably, the pairs Sb328 and Sb330 achieved the highest classification accuracy of 99.84%, suggesting that the EEG features associated with these participants were highly distinctive and readily separable by the model. On the other, this model performance on the pair of sb106 and sb455 were the lowest accuracy score 51.09%.

**Figure 15:**
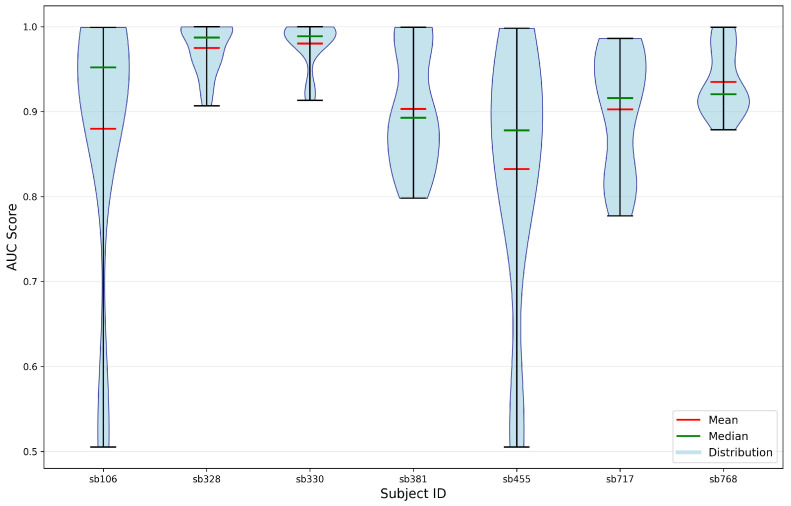
Subject identifiability through uniform DDAE features.

Figure 15 similarly presents subject identifiability based on features extracted from the uniform DDAE. The results, obtained through 5-fold cross-validation, illustrate the distribution of correct identification across all pairwise participant combinations. The analysis indicates that the least identifiable participants in this setting were Sb106 and Sb455.

In uniform DDAE and softmin DDAE, an additional analysis was conducted to further evaluate classification performance. For this purpose, the two participants with the lowest identifiability were excluded from the dataset, and the models were re-assessed using the remaining five participants. This procedure was intended to examine whether the presence of individuals with low discriminability had a negative impact on overall classification accuracy.

**Figure 16:**
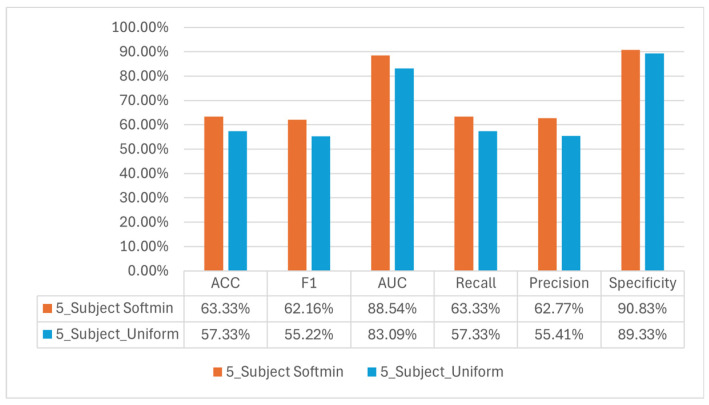
Model performance on five subject identification.

The findings indicate that in both cases, reducing the dataset to five participants led to noticeable changes in model performance. Specifically, conducting classification on a smaller group of participants with more homogeneous and discriminative features allowed for a clearer assessment of the model’s subject identification capability. Accordingly, Figure 16 presents the classification results obtained from five participants under both approaches, providing a comparative view of the models’ performance.

### 5.1. Comparing with the State of the Art

Training and testing biometric models with data from the same or mixed EEG sessions is a common mistake that leads to artificially inflated accuracy scores in EEG-based biometric studies [22]. To properly evaluate a biometric system, longitudinal analysis, which is emphasized by Nakamura et al. [36] and also used in this research, is essential. This was also realized by Pluciska et al., indicating the challenge of a 20% decline in accuracy in accordance with employing cross-session data [37]. Likewise, Kostilek et al. observed a 10% reduction in accuracy when training and testing were conducted on the data from different sessions [38]. Moreover, 79.34% accuracy with SVM was observed in the SEED dataset, which is publicly available for cross-session classification [39]. Other works, applying different pattern classifiers for subject identification, reported accuracies ranging from 82% to 97% [40]. Das et al. proposed a pipeline based on event-related potential (ERP) features, which reached 95% accuracy when trained on data from one session and tested on data from separate sessions [41].

In this study, the highest subject identification accuracy of 100% was through using a longitudinal evaluation, where training and testing data conducted on recordings in separated sessions were obtained through pairwise classification. To the knowledge of the authors, this is one of the novel studies implemented by domain-adaptive autoencoder-based feature extraction on 3D EEG topographic data cubes. Accordingly, this study also provides a unique contribution to the literature by merging methodological rigor with state-of-the-art performance.

Ozdenizci et al. applied deep learning combined with quadratic discriminant analysis (QDA) to a dataset of 10 subjects, achieving an accuracy of 72% [42]. Kostilek et al. employed autoregressive (AR) features with distance-based classification (DBC) for nine subjects and reported 77% accuracy [38]. Maiorana et al. achieved a remarkably low equal error rate (EER) of 2%, using AR features and hidden Markov models (HMM) across 45 subjects [43]. Gonzales et al. integrated AR, fractal complexity coefficients (FCC) and power spectral density (PSD) features with multiple classifiers—such as SVM, KNN, AdaBoost and MLP—reaching up to 73% accuracy with 15 subjects [39]. In contrast, the method proposed in this study leverages DAAE for feature extraction along with various classifiers, yielding promising results, with area under the curve (AUC) scores ranging between 81.06% and 100% in experiments involving seven, five and two subjects.

### 5.2. Limitations and Future Work

It is important to emphasize that this study represents pioneering work in its approach. The impact of the proposed domain adaptation on the effectiveness of EEG feature extraction has been clearly demonstrated. However, the current dataset includes a limited number of subjects and a relatively short time interval between data collection sessions, which may affect the generalizability of the findings over a long duration. Consequently, the proposed methodology can be further validated by using datasets collected over longer time intervals with more subjects.

Other important limitations and practical considerations must be acknowledged. From a computational efficiency perspective, the softmin-based domain generation and autoencoder training introduce additional computational overheads compared to conventional EEG processing pipelines. The iterative optimization of the β parameter and the 3D-to-domain transformation process increase processing time, which may limit real-time applications. Computational complexity scales with the cube resolution and the number of trial signals, potentially requiring high-performance computing resources for large-scale implementations.

Regarding real-world feasibility, the proposed method requires careful consideration of deployment scenarios. The need for sufficient training data to establish reliable domain adaptations may pose challenges in clinical or consumer applications where limited EEG data are available. Moreover, accuracy tends to decline as the number of subjects increases, which is an inherent challenge in biometric identification. This issue could be mitigated by ensuring that there are enough training and testing data to better capture inter-subject variability. Additionally, the method’s performance may be sensitive to variations in EEG acquisition protocols, electrode configurations and environmental conditions that differ from the training setup. The generalization of the ability of different EEG hardware vendors and recording parameters remains to be thoroughly validated. Furthermore, subject-related artifacts such as eye blinks and muscle activities may also influence model performance. To address these concerns, a systematic comparison among heavy, light and no artifact removal strategies can be conducted in future work to further assess and enhance the robustness of the proposed framework.

Privacy implications constitute another critical consideration, particularly for biometric applications. While the domain adaptation process may provide some degree of data anonymization through transformation, the extracted features could potentially retain subject-identifying information. The autoencoder’s latent representations require careful analysis to ensure they do not inadvertently preserve biometric signatures that could compromise user privacy. Furthermore, the computational requirements may necessitate cloud-based processing, raising concerns about data transmission and storage security.

In this study, we applied a ‘light’ artifact removal procedure to preserve the intrinsic signal characteristics and evaluate the robustness of the proposed framework under minimally processed conditions. It is noteworthy that even simple filtering techniques such as the median filter have been demonstrated to effectively reduce artifacts in EEG recordings [44,45]. A systematic comparison between heavy-, light- and no-artifact removal strategies can be considered in future work to further validate the robustness of the proposed framework.

## 6. Conclusions

In this study, we propose an automated framework for feature extraction from EEG data cubes aimed at personal identification. For this purpose, we investigated different types of domain-adaptive autoencoders, presenting two approaches: uniform reference-based domain adaptation and softmin-based domain adaptation. The framework was evaluated under multiple scenarios involving seven, five and two subjects. For the seven-subject classification task, the highest classifier performance in terms of AUC was achieved in the gamma band for resting-state EEG, the De2Be band for auditory stimuli and the Th2Be band for cognitive-task EEG. In pairwise identification, the model successfully distinguished individuals with accuracy ranging from 51.09% to 99.84% by using uniform DDAE features, and from 43.33% to 100% by using softmin DDAE features. After removing the two least identifiable subjects, the five-subject classification task resulted in 57.33% accuracy with uniform DDAE features, and 63.33% with softmin DDAE features. These results indicate that different variants of domain-adaptive autoencoders can lead to notable differences in identification performance, highlighting the impact of feature extraction strategies on EEG-based biometric recognition.

## Figures and Tables

**Table 1:** List of parameters and values.

Model	Parameters
Autoencoder	Activation Functions: [relu, elu, sigmoid, selu]
	Optimizers: [adam, adamW]
	Initializer: [glorot_uniform, glorot_normal, he_uniform, he_normal] Kernel Size: [3 × 3 × 3, 5 × 5 × 3] Number of Layers: [1, 2, 3] Number of Units: [16, 32, 48, 64]
ANN	Activation Functions: [relu, elu, sigmoid, selu] Optimizer: [adam, adamW]
	Initializer: [glorot_uniform, glorot_normal, he_uniform, he_normal] Number of Layers: [1, 2, 3]
SVM	Kernel: [linear, rbf, poly] Gamma: [scale, auto] C: [max_value = 0.1, max_value= 10, sampling = log]
KNN	Number of Neighbors: [3, 4, 5]
	Weights: [uniform, distance] Algorithm: [auto, ball_tree, kd_tree, brute] Leaf Size: [10, 15, 20, 25, 30, 35, 40, 45, 50]
	Distance: [Manhattan, Euclidian]
RF	Number of Estimator: [100, 200, 300] Maximum Depth: [5, 10, 15] Minimum Sample Split: [2, 4, 6, 8, 10]
	Minimum Sample Leaf: [1, 2, 3, 4, 5] Maximum Features: [sqrt, log2]

**Table 2:** Selected parameters for uniform reference DAAE and softmin DAAE.

DDAE Approach	Stimuli-Band	Autoencoder	ANN	KNN	SVM	RF
**Uniform** **Reference**	Resting- Gamma	NE: 200 AF: Relu NL: 4 KI: He_normal KS: 5 × 5 × 3 OPT: Adam NU: 48, 32	NE: 500 AF: Selu NL: 3 KI: He_uniform OPT: AdamW	NN: 4 WGT: distance ALG: auto LS: 30 DSC: Manhattan	KRN: linear GMA: auto C: 0.50	NEST: 100 MD: 10 MSS: 4 MSL: 2 MF: sqrt
**Softmin** **Reference**	Auditory De2Be	NE: 250 AF: Selu NL: 2 KI: Glorot_uniform KS: 5 × 5 × 3 OPT: Adam NU: 16	NE: 400 AF: Relu NL: 2 KI: He_normal OPT: AdamW	NN: 3 WGT: distance ALG: auto LS: 25 DSC: Manhattan	KRN: linear GMA: auto C: 0.50	NEST: 300 MD: 10 MSS: 6 MSL: 3 MF: sqrt

NEs: number of epochs, NLs: number of layers, AF: activation function, KI: kernel initializer, KS: kernel size, OPT: optimizer, NUs: number of units, NNs: number of neighbors, WGTs: weights, ALGs: algorithms, LS: leaf size, DSC: distance, KRN: kernel, GMA: Gamma, NESTs: number of estimators, MD: maximum depth, MSS: minimum sample split, MSL: minimum sample leaf and MFs: maximum features.

## Data Availability

The datasets presented in this article are not readily available because the data are part of an ongoing study. Requests to access the datasets should be directed to omer.soysal@selu.edu.
